# Reducing mother-to-child transmission of HIV: findings from an early infant diagnosis program in south-south region of Nigeria

**DOI:** 10.1186/1471-2458-12-184

**Published:** 2012-03-12

**Authors:** Chukwuemeka Anoje, Bolatito Aiyenigba, Chiho Suzuki, Titilope Badru, Kesiena Akpoigbe, Michael Odo, Solomon Odafe, Oluwasanmi Adedokun, Kwasi Torpey, Otto N Chabikuli

**Affiliations:** 1 FHI 360, Nigeria, Plot 1073 J.S Tarka Street, Area 3 Garki Abuja; 2 Statistics and Monitoring Section, UNICEF, New York, USA; 3 Department of Family Medicine, Medical University of Southern Africa, Pretoria, South Africa

**Keywords:** Mother-to-child transmission of HIV, early infant diagnosis, vertical transmission, breastfeeding, pediatric HIV

## Abstract

**Background:**

Early diagnosis of HIV in infants provides a critical opportunity to strengthen follow-up of HIV-exposed children and assure early access to antiretroviral (ARV) treatment for infected children. This study describes findings from an Early Infant Diagnosis (EID) program and the effectiveness of a prevention of mother-to-child transmission (PMTCT) intervention in six health facilities in Cross-River and Akwa-Ibom states, south-south Nigeria.

**Methods:**

This was a retrospective study. Records of 702 perinatally exposed babies aged six weeks to 18 months who had a DNA PCR test between November 2007 and July 2009 were reviewed. Details of the ARV regimen received to prevent mother-to-child transmission (MTCT), breastfeeding choices, HIV test results, turn around time (TAT) for results and post test ART enrolment status of the babies were analysed.

**Results:**

Two-thirds of mother-baby pairs received ARVs and 560 (80%) babies had ever been breastfed. Transmission rates for mother-baby pairs who received ARVs for PMTCT was 4.8% (CI 1.3, 8.3) at zero to six weeks of age compared to 19.5% (CI 3.0, 35.5) when neither baby nor mother received an intervention. Regardless of intervention, the transmission rates for babies aged six weeks to six months who had mixed feeding was 25.6% (CI 29.5, 47.1) whereas the transmission rates for those who were exclusively breastfed was 11.8% (CI 5.4, 18.1). Vertical transmission of HIV was eight times (AOR 7.8, CI: 4.52-13.19) more likely in the sub-group of mother-baby pairs who did not receive ARVS compared with mother-baby pairs that did receive ARVs. The median TAT for test results was 47 days (IQR: 35-58). A follow-up of 125 HIV positive babies found that 31 (25%) were enrolled into a paediatric ART program, nine (7%) were known to have died before the return of their DNA PCR results, and 85 (67%) could not be traced and were presumed to be lost-to-follow-up.

**Conclusion:**

Reduction of MTCT of HIV is possible with effective PMTCT interventions, including improved access to ARVs for PMTCT and appropriate infant feeding practices. Loss to follow up of HIV exposed infants is a challenge and requires strategies to enhance retention.

## Background

Mother-to-child transmission (MTCT) of HIV is one of the biggest challenges of the HIV/AIDS pandemic especially in resource constrained settings [1,2]. Africa has the highest burden of the disease accounting for about 90 percent of paediatric HIV infections [3]. In 2009 approximately 370,000 children became infected with HIV globally; most of these infections occurred through mother-to-child transmission (MTCT). In the absence of any intervention, the combined risk of mother-to-child transmission of HIV in utero and intra-partum is 15-30 percent. Breastfeeding increases the risk to 20-45 percent [4,5]. Evidence-based research suggests that antiretroviral (ARV) drugs given to pregnant women and their newborn babies reduce the risk of mother-to-child transmission [6-8]. If untreated, HIV infections in children is associated with very high mortality rates [9,10]. A pooled analysis of outcomes of HIV infected infants in sub-Saharan Africa found that 35 percent and 52 percent of HIV-infected children die by age one and two respectively [9].

The empirical evidence of the effectiveness of PMTCT interventions based on short course treatment with antiretroviral drugs has been clearly demonstrated in large scale programs integrated into routine antenatal and obstetrics setting in South Africa and Zambia [11-13].

Early Infant Diagnosis (EID) is part of the infant, maternal, neonatal and child health service package of PMTCT interventions. The DNA Polymerase Chain Reaction (PCR) used for EID tests directly for HIV DNA rather than the HIV antibody and provides definitive diagnosis in children less than 18 months of age [14].

EID makes it possible for HIV-exposed infants to receive early clinical evaluation, prophylaxis for opportunistic infections and antiretroviral therapy (ART) if indicated [15]. Furthermore, a review of HIV test results in an EID program provides a unique opportunity for evaluating the success of the PMTCT program [11].

Nigeria is responsible for 30 percent of the global burden of MTCT of HIV, and is one of the 22 focus countries of the Global Plan to Eliminate MTCT. Though the Government of Nigeria is intensifying PMTCT program efforts to achieve this goal, evidence on the effectiveness of PMTCT interventions or their bottlenecks remains limited [16].

This study examined an Early Infant Diagnosis program in six health facilities in Cross-River and Akwa-Ibom states in South-South Nigeria using routine program data with the aim of evaluating the effectiveness of PMTCT interventions in reducing vertical transmission of HIV and also highlights the challenges of continuum of care for HIV positive babies.

## Methods

### Study design and population

This was a retrospective study. The study population comprised all perinatally HIV exposed children aged six weeks to 18 months who had Dried Blood Spot (DBS) samples taken for a DNA PCR test between November 2007 and July 2009, and for whom a complete set of records were available.

### Study context and site selection

The Government of Nigeria commenced a National EID program in February 2007 [17]. As part of the second phase of the pilot, an EID program was established in six health facilities in Cross River and Akwa Ibom states, South-South Nigeria in November 2007. These sites were supported by the Global HIV/AIDS Initiative in Nigeria (GHAIN), a PEPFAR-funded HIV/AIDS programme implemented by a consortium led by FHI 360. The facilities also had PMTCT and ART programs which were established with support from GHAIN.

Pregnant women registered for antenatal care (ANC) in the study facilities at 14 weeks of gestation and made repeated visits at frequencies determined by their care provider until delivery. During the first ANC visit, all pregnant women received HIV testing and counseling. Following the National PMTCT guidelines at the time of the study [18], pregnant women who tested positive for HIV were evaluated for clinical and immunological eligibility for the commencement of ART. Eligible clients began a first line highly active antiretroviral therapy (HAART) regimen which consisted of a cocktail of either Zidovudine (AZT) or Stavudine (D4T) plus Lamivudine (3TC) plus either Nevirapine (NVP) or Efavirenz (EFZ). The choice of the specific regimen was guided by the clients' clinical condition and presence of contra-indication to any of the drug options. All clients underwent a series of adherence counseling before initiating HAART and on every occasion of a drug refill. HIV positive pregnant women who did not meet the criteria for initiating HAART were placed on HIV chemoprophylaxis, which comprised zidovudine (AZT) beginning at 28 weeks of gestation and administration of single dose nevirapine (sdNVP) during labour. Pregnant women starting prophylaxis at 33 weeks of gestation were given zidovudine (AZT) plus lamivudine (3TC) and sdNVP taken during labour. Women newly diagnosed for HIV during labour received sdNVP alone.

Babies born to HIV positive women received sdNVP within the first three days of life and AZT for six weeks. The HIV-exposed infants were thereafter screened for HIV according to the National EID algorithm which stipulates that a DNA PCR test should be carried out on all HIV-exposed infants at age six weeks. Following the clinic visit for HIV screening, HIV-exposed infants were initiated on cotrimoxazole prophylaxis and their caregivers advised to return in four weeks to receive the DNA PCR test results. The DBS samples collected were couriered to a DNA PCR testing facility (University of Benin Teaching Hospital) located over 600 kilometers from each of the health facilities. The test was carried out using the GeneAmp PCR System 9700 (Perkin-Elmer, Norwalk, Conn, USA). On return of the test results to the facility, caregivers of infants with a positive HIV result were counselled to enrol the infant into HIV care and treatment services, while those who breastfed and whose babies have a negative HIV results were advised to return six weeks after the complete cessation of breastfeeding for a repeat DNA PCR test.

### Data collection and management

This study was based on a review of data routinely collected at six GHAIN-supported EID sites in Cross River and Akwa Ibom states in Nigeria. Routine service data was extracted from structured national data collection tools and entered into a Microsoft Excel spreadsheet^® ^designed for data entry. These national tools from which the data for the analysis were drawn include 1) the PCR request and result forms; 2) the EID register which contain information on the baseline characteristics of HIV-exposed babies, type of ARV chemoprophylaxis received by the mother and the baby, breastfeeding practices, turnaround time and outcome of the DNA PCR test; and 3) the pre-ART register which contains information of HIV positive babies enrolled in HIV care and treatment.

### Data analysis

Data entered into the Microsoft Excel spreadsheet ^® ^was cleaned and checked for consistency. The cleaned data was exported to STATA version 10.0 (Stata Corporation, College Station, TX) for data management and further statistical analysis. Frequency counts were performed to assess completeness of all variables. The DNA PCR result was the major outcome variable in this study and was determined for mother-baby pairs according to ARV prophylaxis received for PMTCT and infant feeding methods. The age-specific transmission rates along with the 95% confidence intervals were estimated for each sub-population. Correlates of mother-to-child transmission of HIV were assessed by multivariable logistic regression. Hosmer and Lemeshow test was used to check for how well the model fit. All tests were two-sided and statistical significance was set at p value < 0.05. From these models, we estimated odds ratios along with 95% confidence intervals, separately for each age group. In addition infants with a positive PCR result were categorized according to their post test ART enrolment status. Differences in proportions were tested using Chi-square test. Fisher's exact test was used to capture associations among categorical variables for frequencies less than 5. The turn around time (TAT) for processing the DBS sample, defined as time (in days) between DBS sample collection and return of DNA PCR results to the facility was described using summary statistics (medians and inter-quartile ranges). Differences in median were tested using median test.

### Ethical considerations

The study was approved by FHI 360's Protection of Human Subjects Committee, North Carolina, USA, and the National Health Research and Ethics Committee (NHREC) Abuja, Nigeria.

## Results

Between November 2007 and July 2009, the DBS samples of 714 HIV exposed babies were sent for their first DNA PCR test. Of these 702 babies were included in the analysis and twelve babies were excluded from the study due to data incompleteness.

### Baseline characteristics

Over half (n = 369) of the study population were males. The median age at the time of DBS sample collection was 13 weeks (Inter-quartile range- 6.5 to 30.5); 70.9% (n = 498) of samples were taken from babies at the age of six weeks or older. Regarding location of DBS sample collection, sizeable proportions (45.9%) of babies were tested in rural facilities. Over one-third of mothers and babies respectively did not receive any ARV medication for preventing mother-to-child transmission of HIV. In relation to infant feeding choices, the vast majority of mothers (79.8%) reported that they breastfed. In particular, 255 (36.3%) reported exclusive breastfeeding and 305 (43.5%) reported mixed feeding (Table 1).

**Table 1:** Baseline characteristics of babies enrolled in the study (N = 702)

*Characteristics*	*Number (%)*
**Gender**	
Male	369 (52.5)
Female	333 (47.5)

**Age at testing**	
≤ 6 weeks	204 (29.1)
6 weeks to 6 months	311 (44.3)
6- 18 months	187 (26.6)

**Maternal ARV**	
HAART	220 (31.3)
AZT + 3TC & single dose NVP in labour	55 (7.8)
AZT & single dose NVP in labour	144 (20.5)
Single dose NVP in labour	13 (1.8)
None	270 (38.5)

**Infant ARV**	
Single dose NVP at birth plus AZT for 4 weeks	434 (61.8)
None	268 (38.2)

**Feeding methods reported at the time of 1^st ^PCR testing**	
Replacement	142 (20.2)
Exclusive breastfeeding	255 (36.3)
Mixed feeding	305 (43.5)

**Facility**	
Rural	322 (45.9)
Urban	380 (54.1)

* Median age 13 weeks (IQR 6.5 to 30.5)

### Age specific transmission rates by intervention and infant feeding status

Analysis of the DNA PCR test results showed that, regardless of infant feeding choice, the transmission rates when both mother and baby received a form of chemoprophylaxis for PMTCT was 4.8% (CI 1.3, 8.3) at zero to six weeks of age and 6.6% (CI 3.0, 10.3) at age six weeks to six months. When neither baby nor mother received an intervention, the transmission rates at age zero to six weeks and six weeks to six months was 19.5% (CI 3.0, 35.5) and 39.8% (CI 29.7, 50.0), respectively. Regardless of chemoprophylaxis, babies who were exclusively breastfed had a transmission rate of 2.7% (CI 0.1, 5.7) at six weeks and 11.8% (CI 5.4, 18.1) from six weeks to six months. Transmission rates among babies whose mothers practised mixed feeding was 13.4% (CI 3.8, 23.1) for babies aged zero to six weeks and 25.6% (CI 18.1, 33.1) for babies aged six weeks to six months (Table 2).

**Table 2:** Age-specific transmission rates by pharmacologic intervention and infant feeding status

Variable	*Number*	*0-6 weeks* *Transmission* *Rate (95% CI)*	*6 wks-6 months* *Transmission* *Rate (95% CI)*	*6-18 months* *Transmission* *Rate (95% CI)*
**Pharmacologic Intervention received**				
Mother and Baby	390	4.8 (1.3, 8.3)	6.6 (3.0, 10.3)	6.4 (0.1, 12.5)
Either Mother or Baby	86	9.3 (1.3, 20.1)	10.8 (3.1, 21.3)	11.7 (5.3,28)
Neither mother nor baby	226	19.5 (3.0, 35.5)	39.8 (29.7, 50.0)	47.7 (38.0, 57.3)

**Infant feeding status**				
Replacement/Formula	142	12.1(1.7, 22.6)	9.2(2.6,15.9)	12.0(1.7,25.7)
Exclusive breastfeeding	255	2.7 (0.1,5.7)	11.8(5.4,18.1)	19.1(6.7,31.4)
Mixed feeding	305	13.4 (3.8,23.1)	25.6(18.1,33.1)	38.3(29.5,47.1)

### Risk of transmission

The unadjusted odds ratio (OR) of MTCT of HIV was 11 times (95% C.I: 6.78-18.35) higher among mother-baby pairs where neither mother nor baby received any ARV compared to mother-baby pairs where both mother and baby received a minimum ARV intervention for PMTCT. When adjusted for the impact of variables such as the sex of the baby, age at first PCR testing, and infant feeding choices, these mother-baby pairs were found to be eight times more likely to transmit HIV infection from mother to baby when compared with babies whose mothers received chemoprophylaxis for PMTCT (AOR 7.76, 95% CI:3.06-10.55). Also from the multivariate analysis, babies who were tested via DNA PCR for the first time between the age of six months to18 months were approximately four times more likely to be infected with HIV than those tested at six weeks of age or less (AOR 3.77, 95% CI: 1.63-8.71) (Table 3).

**Table 3:** Odds ratio of mother to child transmission (N = 702)

*Variable*	*Unadjusted OR(95% C.I)*	*P-value*	*Adjusted OR(95% C.I)*	*p-value*
**Gender**				
Female	1	0.21	1	0.17
Male	1.28(0.87-1.89)		1.36 (0.88-2.12)	

**Age at 1^st ^PCR test**				
≤ 6 weeks	1		1	
6 weeks-6 months	5.58 (2.61-11.95)	< 0.01	1.87 (1.73-8.65)	< 0.01
6-18 months	10.18 (4.69-22.06)	< 0.01	3.77 (1.63-8.71)	< 0.01

**Facility location**				
Urban	1		1	
Rural	1.03 (0.70-1.51)	0.90	1.02 (0.65-1.60)	0.93

**Pharmacologic** **Intervention**				
Mother & baby	1		1	
Mother only	1.68 (0.55-5.11)	0.36	1.54 (0.49-4.84)	0.46
Baby only	2.05 (0.74-5.68)	0.17	1.98 (0.69-5.65)	0.20
None	11.16 (6.78-18.35)	< 0.01	7.76 (4.49-13.49)	< 0.01

**Infant feeding choices** **reported at the time of 1**^**st**^ **PCR test**				
Replacement	1		1	
Exclusive breastfeeding	0.84 (0.42-1.67)	0.62	0.95 (0.44-2.04)	0.90
Mixed feeding	3.38 (1.87-6.10)	< 0.01	1.51 (0.77-2.95)	0.23

### Turnaround time (TAT)

**Figure 1:**
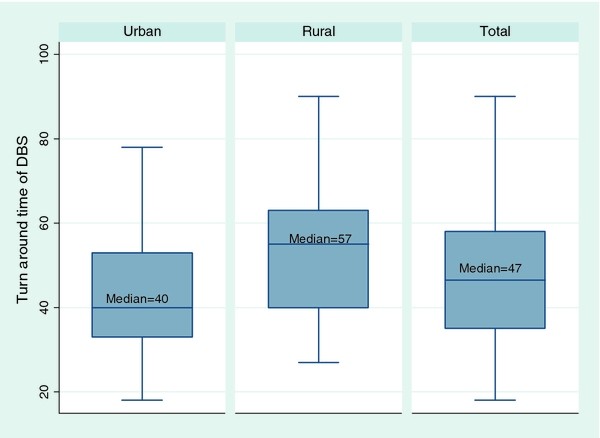
**Box-plot showing median turnaround time (days) of DBS, and by location of facility**.

The median turnaround time (TAT) for processing of DBS samples was 47 days (IQR: 35-58). The median turnaround time in rural settings was 57 days compared to 40 days in urban locations (p < 0.01) (Figure 1).

### Referral to care and treatment

In examining the post test ART enrolment status among the 125 infants with a positive PCR test result, it was observed that 31 babies (25%) were enrolled into a paediatric ART care and treatment program. Nine babies (7%) were known to have died before their DNA PCR results were returned to the facility while records of ART enrolment for the remaining babies (n = 87, 67%) were not located in the six hospitals involved in the study.

## Discussion

For any PMTCT programme to effectively prevent vertical transmission of HIV between mother and baby, a pregnant woman must successfully follow the PMTCT cascade beginning with acceptance of HIV counselling and testing to receiving ARV prophylaxis (if HIV positive) and safe infant feeding practices. Uptake of services provided in this cascade has been shown to be feasible in resource limited settings by studies conducted in Zambia and Ivory Coast where more than 80% of ANC attendees accepted the HIV test and a majority of the HIV positive women commenced ARV prophylaxis even though there were reports of patient attrition between the testing for HIV and commencement of ARV prophylaxis for PMTCT [19,20]. In this study, roughly one-third of the mother-baby pairs did not receive any form of chemoprophylaxis for PMTCT. Patient attrition coupled with a relatively lower ANC attendance rate, 58% in Nigeria [21], is likely to contribute to the suboptimal uptake of ARV prophylaxis for PMTCT observed in this study.

The HIV transmission rate of 4.8% observed for babies between the ages of zero and six weeks where both mother and baby received a pharmacological intervention for PMTCT, and 20% where neither mother nor baby received ARVs, indicates that PMTCT interventions are effective in a resource-limited program setting. These findings compare reasonably with related studies on HIV transmission rates in PMTCT program settings. In Zambia, it was observed that the transmission rates among babies between zero and six weeks old for the two categories of mother-baby pairs mentioned above were 6.5% and 20.9%, respectively [13]. Similarly a study in South Africa by Mnyani et al recorded an overall transmission rate of 5.8% for HIV exposed babies between four and six weeks of age, where both mother and baby received some form of ARVs for PMTCT [22].

In our study, over 80% of the exposed babies screened for HIV were breastfed. This finding is consistent with a related study in Zambia which showed that about 84% of HIV exposed infants whose records were reviewed had ever been breastfed [23]. In addition, our analysis indicated that regardless of pharmacological intervention received by the mother-baby pairs, there was an increase in the age-specific transmission rates for the sub-population of babies who were exclusively breastfed and for those who had mixed feeding. On the other hand, the age-specific HIV transmission rates of babies who were formula fed showed minor changes over time. Prolonged exposure to breastfeeding is likely to have affected HIV transmission rates for babies taking the DNA PCR test at a later date compared with those who had the test at six weeks. Moreover, the observed age specific transmission rates in this study were considerably higher for babies who had mixed feeding compared with those who were exclusively breastfed; this suggests that exclusive breastfeeding is safer than mixed feeding as a feeding option for HIV exposed infants. This finding is consistent with evidence from the ZVITAMBO project in Zimbabwe, which demonstrated that compared with early breastfeeding, early mixed feeding was associated with a four-fold risk of HIV transmission at six months of age for HIV-exposed babies who had previously tested negative at six weeks of age [24].

The 2010, WHO guidelines on HIV and infant feeding recommend that, provided the mother and/or baby is receiving ARVs for their health or as prophylaxis, exclusive breastfeeding should be practiced by HIV-infected mothers for the first six months of life. After the six month period, complimentary feeding should be introduced while continuing with breastfeeding for up to 12 months of age unless replacement feeding is acceptable, feasible, affordable, sustainable and safe for them and their infants before that time [25]. The PMTCT guidelines in Nigeria endorses the WHO guidelines on infant feeding, however the reality is that pregnant women who test positive face a difficult decision about how to feed their babies which is complicated by poor access to proper feeding counselling support and the influence of family members of culturally and socially accepted feeding methods. This ultimately results in improper infant feeding practices as demonstrated by the high rate of mixed feeding practiced by the HIV positive mothers in this study. Accurate information, clear infant feeding guidance, and ongoing support by healthcare workers and family members will help HIV positive mothers succeed with their chosen strategies.

Early diagnosis of HIV in infants provides a critical opportunity to strengthen follow-up of HIV-exposed children and initiate early treatment for those who are HIV infected. If untreated, HIV infection in children is associated with very high mortality rates. The children with HIV early antiretroviral (CHER) study carried out in South Africa showed that early diagnosis and initiation of antiretroviral treatment reduces early infant mortality and HIV progression by 76% and 75% respectively [26]. The national PMTCT guidelines in Nigeria stipulate that the first DNA PCR test should be conducted at six weeks of age for all HIV exposed infants [18]. However, we observed in our study that over 70% of the infants came for the DBS test after six weeks. While acknowledging that the launch of the EID program in these facilities initially resulted in backlog testing of many older age HIV exposed infants, over time it was still observed that majority of HIV exposed infants took the DNA PCR test at ages well over six weeks as stipulated in the PMTCT guidelines. Conducting the DNA PCR tests for early infant diagnosis at six weeks as outlined in the guidelines will offer the health providers the opportunity to strengthen infant feeding counseling provided to the mothers in order to reduce post-natal transmission of HIV.

EID also makes it possible for HIV-exposed infants to receive an early clinical evaluation and be linked to ART care. The finding that only 25% of the 125 babies that tested positive in our study had established evidence of enrolment for paediatric ART at any of the 6 study sites is a cause for concern. The majority of HIV positive babies were not traceable and presumed to be lost to follow up. Given the disease progression and mortality rates in untreated HIV positive infants [10,26], it is reasonable to assume that many of the children lost to follow up had died. However, it was not possible in this study to establish how many babies among those lost to follow up had died. Related studies done in western Malawi also reported high loss to follow up rates for HIV exposed babies and their mothers [27].

Improved client tracking and strengthening the link between the PMTCT, EID and the paediatric ART, coupled with proper and effective counselling of the mothers of HIV-exposed babies before and after the DNA PCR test, will increase the likelihood of enrolling and retaining HIV-exposed babies in ART programs. One model which can be used to improve follow-up for these babies is an integration of HIV-exposed infant follow-up with routine childhood immunisation services. This model is being piloted in Rwanda and preliminary results appear very promising [28]. Expanding the availability of PMTCT services in Primary Healthcare Centres (PHCs) by enabling access to CD4 count test through a 'hub and spoke' laboratory referral system and the provision of short course ARVs for PMTCT at PHCs is an option that is worth exploring. The feasibility of this strategy has been demonstrated in Zambia even though completion of the CD4 count assessment at the PHCs remains a challenge [29]. The proposed strategy also has the potential to minimize patient attrition as PHCs are generally more accessible to patients.

The delay in establishing a diagnosis is another factor that likely contributed to the high attrition rate observed in this study. Though the threshold turnaround time (TAT) for the return of lab results to the DBS collection centers in the national EID program is 28 days, in this study the median TAT for processing of DBS samples was almost double this figure (47 days). A shorter TAT will minimise the frustration and anxiety caregivers face while waiting for the HIV results. This will ultimately reduce loss to follow up and provide an opportunity for early initiation for ART.

The limitations to our study include the purposive nature of the selection of sites that offered EID services in the two states where the study was carried out. In addition, use of facility based records may have led to selection bias (i.e., study population included those who sought services at facilities). These limitations affected our ability to generalize the findings of this study. Additionally, some findings have limited statistical significance due to a limited sample size. Furthermore, we acknowledge that our analysis would have been strengthened by including variables such as baseline CD4 counts and clinical staging, however, the selection of the variables included in our analysis was guided (and thus limited) by what we were able to obtain from the national PMTCT data collection tools used and maintained at the study sites.

## Conclusion

The findings of this study demonstrate that reduction in maternal-to-child transmission of HIV is possible with effective PMTCT intervention. Increase in the uptake of chemoprophylaxis and ensuring appropriate infant feeding practices is critical in reducing vertical transmission of HIV. Strategies to address programmatic challenges of long turnaround time, effective referrals and linkage to care are essential. PMTCT programs with EID should be encouraged to conduct similar analyses to determine the effectiveness and bottlenecks in their interventions.

## Competing interests

The authors declare that they have no competing interests.

## Authors' contributions

CA, MO and OC conceived the study, CA, MO, OC, BA, OA and SO participated in the study design. CA, MO, KA, OA coordinated data collection. CA, BA, SO, OA, CS, OC, KT drafted the article. TB, KA, and CA performed the statistical analysis. CS, KT and OC provided critical review of the article. All authors read and approved the final manuscript.

## Pre-publication history

The pre-publication history for this paper can be accessed here:

http://www.biomedcentral.com/1471-2458/12/184/prepub
